# Molecular Components of Store-Operated Calcium Channels in the Regulation of Neural Stem Cell Physiology, Neurogenesis, and the Pathology of Huntington’s Disease

**DOI:** 10.3389/fcell.2021.657337

**Published:** 2021-04-01

**Authors:** Ewelina Latoszek, Magdalena Czeredys

**Affiliations:** Laboratory of Neurodegeneration, International Institute of Molecular and Cell Biology in Warsaw, Warsaw, Poland

**Keywords:** store-operated Ca^2+^ entry, store-operated Ca^2+^ channels, Ca^2+^ homeostasis, neural stem cells, induced pluripotent stem cells, brain organoids, Huntington’s disease

## Abstract

One of the major Ca^2+^ signaling pathways is store-operated Ca^2+^ entry (SOCE), which is responsible for Ca^2+^ flow into cells in response to the depletion of endoplasmic reticulum Ca^2+^ stores. SOCE and its molecular components, including stromal interaction molecule proteins, Orai Ca^2+^ channels, and transient receptor potential canonical channels, are involved in the physiology of neural stem cells and play a role in their proliferation, differentiation, and neurogenesis. This suggests that Ca^2+^ signaling is an important player in brain development. Huntington’s disease (HD) is an incurable neurodegenerative disorder that is caused by polyglutamine expansion in the huntingtin (HTT) protein, characterized by the loss of γ-aminobutyric acid (GABA)-ergic medium spiny neurons (MSNs) in the striatum. However, recent research has shown that HD is also a neurodevelopmental disorder and Ca^2+^ signaling is dysregulated in HD. The relationship between HD pathology and elevations of SOCE was demonstrated in different cellular and mouse models of HD and in induced pluripotent stem cell-based GABAergic MSNs from juvenile- and adult-onset HD patient fibroblasts. The present review discusses the role of SOCE in the physiology of neural stem cells and its dysregulation in HD pathology. It has been shown that elevated expression of STIM2 underlying the excessive Ca^2+^ entry through store-operated calcium channels in induced pluripotent stem cell-based MSNs from juvenile-onset HD. In the light of the latest findings regarding the role of Ca^2+^ signaling in HD pathology we also summarize recent progress in the *in vitro* differentiation of MSNs that derive from different cell sources. We discuss advances in the application of established protocols to obtain MSNs from fetal neural stem cells/progenitor cells, embryonic stem cells, induced pluripotent stem cells, and induced neural stem cells and the application of transdifferentiation. We also present recent progress in establishing HD brain organoids and their potential use for examining HD pathology and its treatment. Moreover, the significance of stem cell therapy to restore normal neural cell function, including Ca^2+^ signaling in the central nervous system in HD patients will be considered. The transplantation of MSNs or their precursors remains a promising treatment strategy for HD.

## Introduction

Store-operated Ca^2+^ entry (SOCE) is the process by which calcium (Ca^2+^) flows from the extracellular space into the cytoplasm in response to the depletion of endoplasmic reticulum (ER) Ca^2+^ stores. Upon a decrease in Ca^2+^ in the ER, Ca^2+^ sensors, named stromal interaction molecules (STIMs; e.g., STIM1 and STIM2) (Liou et al., 2005; Roos et al., 2005; Zhang et al., 2005) interact with highly selective Orai1-3 Ca^2+^ channels at the plasma membrane (Feske et al., 2006; Peinelt et al., 2006; Prakriya et al., 2006), which causes Ca^2+^ influx. The current mediated by Orai channels is called Ca^2+^ release-activated Ca^2+^ current (I_CRAC_) and is highly selective for Ca^2+^ (Hoth and Penner, 1993; Lewis and Cahalan, 1995; Moccia et al., 2015). Besides, there also exists store-operated Ca^2+^ current (I_SOC_), which is characterized by non-selective outward currents that are operated by STIM and transient receptor potential canonical 1 (TRPC1) channel (Golovina et al., 2001; Trepakova et al., 2001; Strübing et al., 2003; Ma et al., 2015; Majewski and Kuznicki, 2015; Lopez et al., 2016; Lopez et al., 2020). SOCE has been detected in both non-excitable cells (Feske et al., 2005; Parekh and Putney, 2005; Prakriya and Lewis, 2015; Putney et al., 2017) and neurons (Emptage et al., 2001; Baba et al., 2003; Klejman et al., 2009; Gemes et al., 2011; Gruszczynska-Biegala et al., 2011; Wu et al., 2011, 2016; Hartmann et al., 2014; Samtleben et al., 2015; Czeredys et al., 2017). Recent research has shown that Ca^2+^ signaling, including the SOCE process has been involved in the physiology of neural stem cells (NSCs) and neurogenesis (Tonelli et al., 2012; Toth et al., 2016; Glaser et al., 2019).

Stem cells are unspecialized cells that are characterized by their ability to self-renew to increase their pool. They also have the potential to differentiate (specialize) into specific types of cells (e.g., neuronal cells) (Hima Bindu and Srilatha, 2011; Tonelli et al., 2012; Zakrzewski et al., 2019). NSCs represent a population of multipotent cells, which can self-renew and proliferate without limitation, and finally, differentiate into neurons, astrocytes, and oligodendrocytes (Oikari et al., 2016; Golas, 2018). In contrast, neural progenitor cells (NPCs) are also multipotent and can differentiate into more than one cell type, but have a limited ability to proliferate and are unable to self-renew (Oikari et al., 2016; Golas, 2018). However, Golas, note that researchers use the term NSCs and NPCs interchangeably (Golas, 2018). NSCs/NPCs are present in fetal neural tissue, but also neonatal and adult brain (Yin et al., 2013; Martínez-Cerdeño and Noctor, 2018; Obernier and Alvarez-Buylla, 2019). The major stem cell niches in the adult brain are NSCs in the subgranular zone (SGZ) of the hippocampal dentate gyrus and subventricular zone (SVZ), which is situated on the outside wall of each lateral ventricle of the vertebrate brain (Ma et al., 2009). In these regions, NSCs can proliferate, self-renew, and give birth to both neurons and glial cells. In adulthood, neurogenesis continues to occur throughout life (Galvan and Jin, 2007).

Stem cells can differentiate into different specialized, mature cell types. There are five types of stem cell development potential, which can be arranged hierarchically. The first type is totipotency, in which cells differentiate into embryonic and extra-embryonic cell (EC) types. Among these are the fertilized egg cell, the zygote. Zygotes have the highest differentiation potential because they can create a whole, complete organism (Hima Bindu and Srilatha, 2011; Zakrzewski et al., 2019). The second type are pluripotent cells. These cells can differentiate into any somatic cell, forming cells of all germ layers. However, they cannot transform into trophoblast cells (Hima Bindu and Srilatha, 2011; Zakrzewski et al., 2019). This type of cell includes embryonic stem cells (ESCs) and induced pluripotent stem cells (iPSCs). Dental pulp stem cells (DPSCs) and stem cells from human exfoliated deciduous teeth (SHEDs) can differentiate into several lineages, such as neurons, hepatocytes, endothelial cells, and odontoblasts (Rosa et al., 2016). The third type is multipotent cells, which are present during embryo development in the gastrula stage, consisting of mesoderm, endoderm, and ectoderm cells. They can differentiate into cells that belong to specific tissue. One example is adult NSCs, which can develop mature neurons and glial cells and have an ectodermal origin (Ma et al., 2009; Tonelli et al., 2012). Additionally, multipotent cells are hematopoietic stem cells, which have a mesodermal origin and can develop into several types of blood cells (Zakrzewski et al., 2019). Lung cells derive from lung endodermal progenitor cells (Parekh et al., 2020). The next type is oligopotent stem cells, which can differentiate into a few cell types. One example is myeloid stem cells, which can divide into white blood cell lineages (Hima Bindu and Srilatha, 2011; Zakrzewski et al., 2019). In turn, unipotent cells have lower potency and can develop only one specific cell type (e.g., muscle stem cells that give rise to mature muscle cells) (Hima Bindu and Srilatha, 2011). The fate of stem cells is determined by factors in the cell environment and tissue-specific niches, such as growth factors and neurotrophins (e.g., transforming growth factor β [TGF-β], fibroblast growth factor [FGF], and nerve growth factor [NGF]), cytokines (activin A), and other signaling molecules (Xiao and Le, 2016; Tan et al., 2019). One of them is Ca^2+^, which plays a critical role in various stages of stem cell differentiation as a component of signaling pathways (Tan et al., 2019).

Huntington’s disease (HD) is a heritable neurodegenerative disorder that is characterized by unintentional movements, cognitive decline, and behavioral impairment. A mutant form of huntingtin (mHTT) protein contains an expansion of polyglutamine residues (polyQ) in its amino-terminal part that causes its aggregation (Ross, 2002) and the loss of γ-aminobutyric acid (GABA)-ergic medium spiny neurons (MSNs) in the striatum (Vonsattel and DiFiglia, 1998; Zoghbi and Orr, 2000). When mHTT has between 39 and 60 glutamine repeats, late-onset HD begins, usually at 30–50 years of age. Early HD onset characterizes the juvenile form of HD, in which disease progression occurs before the age of 21, and the polyQ chain contains over 60 glutamine repeats (Quigley, 2017). Huntington’s disease is also considered a neurodevelopmental disorder (Wood, 2018; Lebouc et al., 2020).

Recent research has shown the transcriptional dysregulation of several genes, including genes that are involved in the calcium signalosome in the striatum in YAC128 mice (i.e., a mouse model of HD) (Czeredys et al., 2013). SOCE was shown to be significantly elevated in several models of HD, including cellular models (Vigont et al., 2014, 2015) and MSNs from the YAC128 mice model (Wu et al., 2011, 2016; Czeredys et al., 2017). Furthermore, patch-clamp recordings revealed that I_SOC_ increased in cellular HD models (Wu et al., 2011; Vigont et al., 2015). STIM2 plays a key role in the dysregulation of SOCE in YAC128 mice. An increase in STIM2 expression was observed in both aged MSN cultures and in the striatum in YAC128 mice (Wu et al., 2016). Abnormal synaptic neuronal SOCE (nSOCE) likely underlies synaptic loss in MSNs (Wu et al., 2016; Wu J. et al., 2018). The knockdown of molecular components of SOCE, including the Ca^2+^ sensor STIM2 and Ca^2+^ channels TRPC1, TRPC6, Orai1, and Orai2, resulted in the stabilization of dendritic spines and restored elevations of nSOCE in YAC128 MSNs (Wu et al., 2016; Wu J. et al., 2018). Although a few compounds have been shown to stabilize elevations of SOCE, such as tetrahydrocarbazoles (Czeredys et al., 2017, 2018) and the quinizone derivative EVP4593 (an NF-κB antagonist which restores synaptic nSOCE and rescues abnormal spines in YAC128 MSNs *in vitro* and *in vivo*) (Wu et al., 2011, 2016), future research is needed to determine whether they can serve as drug candidates for HD therapy (Czeredys, 2020). No effective treatments are currently available for HD. One option for HD patients could be stem cell therapy, but this approach is still under investigation and many issues must be improved to increase the reliability of transplants (Bachoud-Lévi et al., 2020). It is difficult to obtain a genuine MSN fate, therefore further advances are required to achieve suitable donor cells for replacement therapy for HD.

The present review focuses on Ca^2+^ signaling via store-operated Ca^2+^ channels under physiological conditions in NSC development and the pathology of HD. The role of STIM, Orai, and TRPC proteins in NSC proliferation and differentiation and neurogenesis is discussed. The dysregulation of SOCE that has been detected in iPSC-based GABAergic MSNs from juvenile- and adult-onset HD patient fibroblasts and its contribution to HD pathology is also presented. We discuss advances in the application of established protocols to obtain MSNs from iPSCs and other stem cell sources. We also discuss recent progress in brain organoid technology and its potential use for examining HD pathology and treatment. Finally, we summarize the latest findings on the transplantation of precursors of MSNs into HD patient brains. Stem cell therapy is a promising treatment strategy for HD.

## Ca^2+^ Signaling Via Store-Operated Ca^2+^ Channels in NSCs Under Physiological Conditions

Ca^2+^ signaling is a crucial player in early neural development, which is distinguished by the fast proliferation of ECs, which then differentiate to produce many specialized cell types, including neurons. One of the major pathways of Ca^2+^ entry in non-excitable cells, such as NPCs, is SOCE (Shin et al., 2010). In human embryonic stem cells (hESCs), SOCE but not voltage-gated Ca^2+^ channel (VGCC)-mediated Ca^2+^ entry is detected (Huang et al., 2017). SOCE has been recently measured using Fura-2-acetoxymethyl ester (Fura2-AM) in human neural progenitor cells (hNPCs) and spontaneously differentiated neurons that derive from pluripotent hESCs (Gopurappilly et al., 2019). In embryonic and adult mouse NSCs/NPCs from the ganglionic eminence (GE) and anterior SVZ, respectively, SOCE was mediated by Ca^2+^ release-activated Ca^2+^ (CRAC) channel proteins STIM1 and Orai1. The knockdown of STIM1 or Orai1 significantly decreased SOCE in NPCs *in vitro*. Moreover, SOCE was lost in NPCs from transgenic mice that lacked Orai1 or STIM1 and in knock-in mice that expressed a loss-of-function Orai1 mutant (R93W). In NPCs, the SOCE process was initiated by epidermal growth factor (EGF) and acetylcholine the latter of which involved the contribution of muscarinic receptors. CRAC channels regulated calcineurin/nuclear factor of activated T cell (NFAT)-mediated gene expression. The inhibition or ablation of STIM1 and Orai1 expression significantly attenuated the proliferation of embryonic and adult NPCs that were cultured as neurospheres and also *in vivo* in the SVZ in adult mice. This observation indicated that CRAC channels are crucial determinants of mammalian neurogenesis (Somasundaram et al., 2014). Ca^2+^ entry through SOCE, regulated by Orai channels in hNPCs and neurons that differentiated from hNPCs, was shown to be negatively regulated by septin 7 (SEPT7), a protein that is a member of the family of filament-forming guanosine triphosphatases, called septins (Deb et al., 2020).

To understand the role of SOCE in human NSC physiology, Gopurappilly et al. (2018) knocked down STIM1 in hNPCs. These cells were characterized by an efficient SOCE process that was significantly reduced by STIM1 knockdown. The global transcriptomic approach of STIM1-knockdown hNPCs indicated the downregulation of genes that are related to cell proliferation and DNA replication processes, whereas genes that are related to neural differentiation, including postsynaptic signaling, were upregulated. Additionally, STIM1-knockdown NPCs substantially attenuated the average size of neurospheres and their numbers. In parallel, they exhibited spontaneous differentiation into a neuronal lineage. These findings indicate that gene expression that is modulated by STIM1-mediated SOCE is responsible for the regulation of self-renewal and the differentiation of hNPCs. The authors considered that the loss of SOCE *in vivo* could result in the attenuation of an appropriate number of hNPCs that are needed for normal brain development (Gopurappilly et al., 2018). Additionally, Pregno et al. (2011) showed that the neuregulin-1/Erb-B2 receptor tyrosine kinase 4 (ErbB4)-induced migration of ST14A striatal progenitors cells was modulated by *N*-methyl-D-aspartate receptor (NMDAR) activation in a Ca^2+^-dependent manner that was made possible by SOCE.

In mouse ESCs, both SOCE components STIM1 and Orai1 were expressed and functionally active (Hao et al., 2014). Their knockdown reduced SOCE in ECs. STIM1 and Orai1 expression levels were enhanced during neural differentiation. STIM1 was suggested to play an important role in the early stage of neural lineage entry. In contrast to Hao et al. (2014), Gopurappilly et al. (2018) observed that STIM1 and STIM2, but not Orai1, were shown to be involved in the early neural differentiation of ESCs. Moreover, STIM1 knockdown resulted in substantial cell loss and blocked the proliferation of neural progenitors that were derived from mouse ESCs. Hao et al. (2014) concluded that inhibition of the neural differentiation of mouse ESCs by STIM1 and STIM2 knockdown is independent of Orai1-mediated SOCE. Additionally, STIM1 is involved in the terminal differentiation of mouse neural progenitors into neurons and astrocytes (Hao et al., 2014). Furthermore, upregulation of the Stim1b isoform was detected in differentiating cells compared with cells that underwent proliferation in zebrafish neurospheres (Tse et al., 2018).

In mouse SVZ cells, the expression of TRPC1 and Orai1 channels and their activator STIM1 was reported, and these cells were characterized by functional SOCE (Domenichini et al., 2018). The application of SOCE inhibitors *in vitro* (SKF-96365 or YM-58483) decreased the stem cell population by attenuating their proliferation and dysregulating SVZ stem cell self-renewal by driving their asymmetric division instead of symmetric proliferative division. Domenichini et al. (2018) detected TRPC1, Orai1, and STIM1 expression in mouse brain sections *in vivo* in sex-determining region Y-box2 (SOX2)-positive SVZ NSCs. The inhibition of SOCE reduced the population of stem cells in the adult mouse brain and impaired the ability of SVZ cells to create neurospheres *in vitro*. These results indicated that SOCE plays a key role in the regulation of NSC activation and self-renewal (Domenichini et al., 2018).

Several studies established a role for TRPC channels in NSC regulation. Ca^2+^ entry through TRPC1 channels appears to play a critical role in basic fibroblast growth factor (bFGF)-induced NSCs proliferation (Fiorio Pla et al., 2005). TRPC1 and TRPC4 modulate neurite extension in hESCs (Weick et al., 2009), whereas they regulate their proliferation in oligodendrocyte precursor cells (Paez et al., 2011). Additionally, Li et al. (2012) reported the role of TRPC1 and SOCE in adult hippocampal neurogenesis. The TRPC1-mediated elevation of Ca^2+^ entry was shown to be essential for the proliferation of adult hippocampal NPCs. Upon TRPC1 knockdown, the induction of cell cycle arrest in the G0/G1 phase was observed and caused the up- or downregulation of 10 cell cycle genes, indicating that these genes may regulate the effects of TRPC1 on adult NPCs proliferation. Antagonists of SOCE and canonical TRPC inhibited the increase in SOCE and adult NPC proliferation. Moreover, the knockdown of Orai1 and STIM1 inhibited SOCE and proliferation in adult NPCs (Li et al., 2012). The role of TRPCs as SOCE channels was also examined with regard to modulating induction of the neuronal differentiation of NPCs with a neural cell surface antigen of A2B5 (A2B5+ NPCs) that were obtained from the postnatal day 12 rat brain. In neural cells that differentiated from A2B5+ NPCs, SOCE substantially increased compared with proliferating cells, and the application of SOCE inhibitors normalized these processes. Pharmacological inhibitors of SOCE and an siRNA against TRPC5 normalized the amplitude of SOCE and inhibited neural differentiation from A2B5+ NPCs (Shin et al., 2010). TRPC3 was recently shown to play an important role in the survival, pluripotency, and neural differentiation of mouse embryonic stem cells (mESCs) (Hao et al., 2018). The CRISPR/Cas9-facilitated knockout of TRPC3 caused apoptosis and compromised mitochondrial membrane potential in undifferentiated mESCs and neurons during the course of differentiation. Furthermore, TRPC3 knockout impaired the pluripotency of mESCs and strongly attenuated the neural differentiation of mESCs by inhibiting the expression of genes that encode neural progenitors, neurons, astrocytes, and oligodendrocytes (Hao et al., 2018).

**FIGURE 1:**
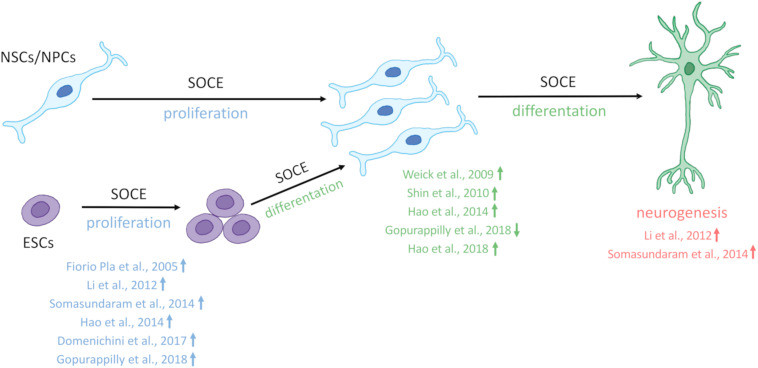
SOCE regulates neural stem cell physiology. Store-operated Ca^2+^ entry (SOCE) and its molecular components positively regulate the proliferation of embryonic stem cells (ESCs) and neural stem cell (NSCs)/neural progenitor cells (NPCs), differentiation of ECSs, and NSCs/NPCs as well as neurogenesis. References that correspond to respective discoveries are shown in the figure. The upward arrows indicate that SOCE and its molecular players positively regulate proliferation, differentiation, and neurogenesis. Compared with other studies Gopurappilly et al. (2018) (reference indicated in the figure by a down arrow) detected a decrease in NSC differentiation.

SOCE is crucial for the physiology of NSCs and regulates Ca^2+^ signaling in newly developing neurons in the brain (Figure 1). The main molecular components of SOCE that directly regulate Ca^2+^ signaling in NSC development and their main physiological functions are listed in Table 1.

**TABLE 1:** Molecular components of SOCE in neural stem cell physiology.

Molecular component of SOCE	Function in neural stem cell physiology	References
**STIM1,** **ORAI1**	- Their inhibition or removal attenuated the proliferation of embryonic NPCs cultured as neurospheres *in vitro.* - Their inhibition or removal attenuated the proliferation of adult NPCs that were cultured as neurospheres *in vitro* and *in vivo* in the SVZ of adult mice.	Somasundaram et al., 2014
**STIM1**	- Its knockdown in hNPCs caused the downregulation of genes that are involved in cell proliferation and DNA replication and the upregulation of genes that are involved in neural differentiation. - Its knockdown in NPCs attenuated neurospheres size and number. - STIM1 knockdown increased spontaneous differentiation into the neuronal lineage.	Gopurappilly et al., 2018
**STIM1,** **ORAI**	- STIM1 and Orai1 expression levels increased during neural differentiation. - STIM1 plays a role in the early stage of neural lineage entry. - STIM1 and STIM2 but not Orai1 are involved in the early neural differentiation of ES cells. - STIM1 knockdown caused severe cell loss and inhibited the proliferation of neural progenitors that were derived from mouse ESCs. - STIM1 or STIM2 knockdown in EC cells is independent of Orai1-mediated SOCE. - STIM1 is involved in the terminal differentiation of mouse ESCs into neurons and astrocytes.	Hao et al., 2014
**STIM1**	- Upregulation of the Stim1b isoform was shown in differentiating cells compared with those that underwent proliferation in zebrafish neurospheres.	Tse et al., 2018
**TRPC1, Orai1, STIM1**	- Mouse SVZ cells express TRPC1, Orai1, and STIM1. - TRPC1, Orai1, and STIM1 are expressed in mouse brain sections *in vivo* in SOX2-positive SVZ NSCs. - Application of SOCE inhibitors *in vitro* (SKF-96365 or YM-58483) decreased the stem cell population by attenuating their proliferation and dysregulating SVZ stem cell self-renewal. - Inhibition of SOCE reduced the population of stem cells in the adult mouse brain and impaired the ability of SVZ cells to create neurospheres *in vitro*.	Domenichini et al., 2018
**TRPC1**	- Ca^2+^ entry through these channels regulates bFGF-induced NSC proliferation.	Fiorio Pla et al., 2005
**TRPC1, TRPC4**	- They modulate neurite extension in hESCs.	Weick et al., 2009
**TRPC1,** **ORAI,** **STIM1**	- TRPC1-mediated elevation of Ca^2+^ entry plays a role in adult hippocampal neurogenesis. - TRPC1-mediated elevation of Ca^2+^ entry is essential for the proliferation of adult hippocampal NPCs. - TRPC1 knockdown induces cell cycle arrest in the G0/G1 phase and dysregulates genes that are involved in cell cycle regulation. - Antagonist inhibition of TRPC1-mediated elevation of Ca^2+^ entry inhibited adult NPC proliferation. - Knockdown of Orai1 or STIM1 inhibited SOCE and proliferation in adult NPCs.	Li et al., 2012
**TRPC5**	- Pharmacological inhibition of SOCE and siRNA against TRPC5 blocked neural differentiation from A2B5+ NPCs.	Shin et al., 2010
**TRPC3**	- Plays a role in the survival, pluripotency, and neural differentiation of mESCs. - TRPC3 knockout caused apoptosis and disrupted mitochondrial membrane potential in both undifferentiated mESCs and neurons that were undergoing differentiation. - TRPC3 knockout impaired the pluripotency of mESCs and attenuated the neural differentiation of mESCs.	Hao et al., 2018

## Molecular Components of Soce and Ca^2+^ Signaling Pathways Might Contribute to Neurodevelopmental Changes in HD

Increasing evidence from iPSCs and a three-dimensional (3D) organoid model of HD indicates that HD is also a neurodevelopmental disease (Conforti et al., 2018; Świtońska et al., 2018; Wiatr et al., 2018). Neural cells that were differentiated from juvenile HD patient-derived iPSC lines exhibited deficits in neurodevelopment and adult neurogenesis. RNA sequencing (RNAseq) analyses of these cells showed that one-third of the genes that were altered are involved in the regulation of neuronal development and maturation (HD iPSC Consortium., 2017). Additionally, in neurons that were differentiated from iPSCs from juvenile HD patients, RNAseq revealed the upregulation of several genes that encode proteins that are involved in Ca^2+^ signaling, including inositol-1,4,5-triphosphate receptor 1 (IP3R1), TRPC6, and CRAC channels, and the downregulation of genes that encode proteins that are involved in cyclic adenosine monophosphate (cAMP) response element-binding protein (CREB), glutamate, and GABA signaling, axonal guidance, and synaptic function (HD iPSC Consortium., 2017). Transcriptomic analysis of juvenile-onset HD found that iPSC-derived NSCs had significantly different expression levels of genes that are involved in signaling, the cell cycle, axonal guidance, and neuronal development compared with controls, whereas NSCs that contained medium-length polyQ tracts were characterized by changes in Ca^2+^ signaling (HD iPSC Consortium., 2012). The dysregulation of several Ca^2+^ signalosomes that are involved in Ca^2+^ binding and Ca^2+^ signaling were identified in iPSC-based GABAergic MSNs from late-onset HD patient fibroblasts (Nekrasov et al., 2016). These findings corroborate the importance of Ca^2+^ dysregulation in neurodevelopmental changes that are observed in HD pathology. The treatment of neural stem or progenitor cells with isoxazole-9, which increases Ca^2+^ influx via NMDARs and VGCCs, was neuroprotective in juvenile- and adult-onset iPSC-derived neurons and restored cortico-striatal synapses in R6/2 mice, a model of HD (HD iPSC Consortium., 2017). In rat cortical neuronal cultures, STIM proteins negatively regulate NMDA (Gruszczynska-Biegala et al., 2020) and STIM1 inhibits L-type VGCCs (Park et al., 2010; Wang et al., 2010; Dittmer et al., 2017). Therefore, the elevation of SOCE might affect these receptors in HD.

Additionally, neuronal differentiation is delayed in iPSC-derived MSNs from juvenile HD patients (Mathkar et al., 2019). Differentiated HD NPCs had a higher number of the stem cell marker Nestin on days 14, 28, and 42, however, no increased proliferation was observed. Both the knockdown of mHTT and inhibition of the Notch pathway stabilized Nestin expression in these cells (Mathkar et al., 2019). Recent data also clearly showed that HD has a neurodevelopmental component and thus is not simply a neurodegenerative disease (Barnat et al., 2020). In the developing cortex from fetal tissues that carried mHTT with 39, 40, and 42 polyQ repeats, which can lead to manifestations of adult-onset HD, Barnat et al. identified several cellular abnormalities. These changes included the abnormal localization of mHTT and junctional complex proteins, disruptions of neuroprogenitor cell polarity and differentiation, improper ciliogenesis, and defects in mitosis and cell cycle progression. Additionally, mHTT was shown to attenuate cell proliferation and shift neurogenesis toward the neuronal lineage (Barnat et al., 2020).

## Dysregulation of Soce in iPSC-Derived Models From HD Patients

In recent studies, iPSC-based GABAergic MSNs from HD patient fibroblasts that exhibit progressive HD pathology *in vitro* were generated by several groups (An et al., 2012; Jeon et al., 2012; Nekrasov et al., 2016). Nekrasov et al. (2016) reported that iPSC-based GABAergic MSN neurons from HD patient fibroblasts (40–47 CAG repeats) representing adult-onset HD manifested progressive HD phenotype, including mHTT aggregation, an increase in the number of phagosomes, and an increase in neural death overtime. They also observed that these neurons were characterized by dysregulated SOCE what was measured using the patch-clamp technique (Nekrasov et al., 2016). In HD iPSC-based GABAergic MSNs, SOC currents were shown to be mediated by I_CRAC_ and I_SOC_, which were upregulated simultaneously compared with wildtype iPSC-based GABAergic MSNs (Vigont et al., 2018). The molecular mechanism by which SOCE is elevated in MSNs from adult-onset HD fibroblasts is unrevealed. Transcriptome analysis has been previously demonstrated that the expression of genes encoding Orai and TRP channels and STIM proteins did not differ significantly between iPSCs-derived MSN cultures compared to control and their protein levels were not further studied (Nekrasov et al., 2016). Furthermore, the SOCE inhibitor EVP4593 stabilized I_SOC_ and I_CRAC_ in HD iPSC-based GABAergic MSNs (Vigont et al., 2018). The mechanism of action of EVP4593 is still unknown, but Vigont et al. (2018) suggested that this compound may target SOCE regulatory proteins (e.g., STIM proteins) that are involved in both I_CRAC_ and I_SOC_ because EVP4593 equally affected both I_CRAC_ and I_SOC_. Besides EVP4593 attenuated the number of phagosomes and exerted neuroprotective effects during neuronal aging (Nekrasov et al., 2016).

A recent publication by Vigont et al. (2021) indicates that Ca^2+^ signaling is highly elevated in iPSC-based GABAergic MSNs cultures from juvenile-onset HD patient containing 76 repetitions of CAG in mHTT. Authors specifically demonstrated higher levels of store-operated and voltage-gated calcium uptakes in these cells using the patch-clamp technique. Upregulation of Ca^2+^ sensor, STIM2 was detected in juvenile-onset HD MSNs, and shRNA-mediated suppression of STIM2 attenuated SOCE, but not affected VGCC. Moreover, G418, which is a known selective antagonist of STIM2 (Parvez et al., 2008) decreased SOCE in these cells. Vigont et al. (2021) concluded that elevated expression of STIM2 underlies the excessive Ca^2+^ entry through nSOCs in juvenile-onset HD pathology. Additionally, the neuroprotective effect of SOCE inhibitor, EVP4593 was shown in the stabilization of high protein levels of both total HTT and STIM2 via regulation of their expression (Vigont et al., 2021). Interestingly, the severity of Ca^2+^ influx via SOCE was independent of CAG repeat length between juvenile- and adult-onset HD iPSCs-derived MSNs (Vigont et al., 2021). Additionally, a similar increase of voltage-gated calcium uptakes was observed in both juvenile- and adult-onset HD iPSCs-derived MSNs (Vigont et al., 2021).

**FIGURE 2:**
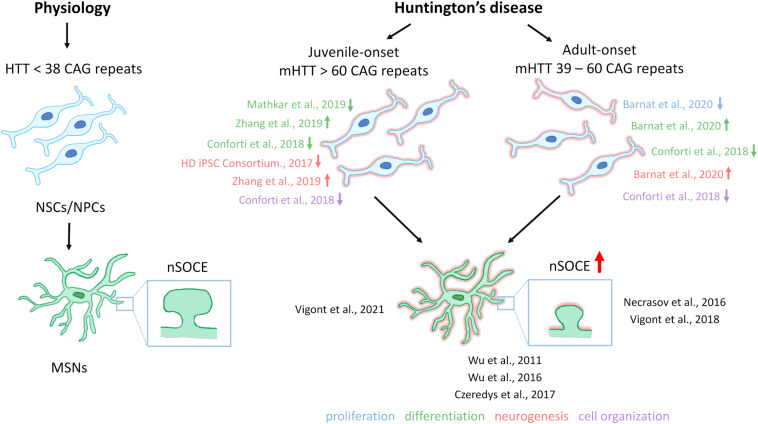
Neural stem cells in HD pathology and contribution of SOCE to HD MSN pathology. Under physiological conditions, neural stem cells (NSCs)/neural progenitor cells (NPCs) are characterized by proper cell organization and development. The dysfunction of NSCs/NPCs under conditions of HD pathology, including both juvenile- and adult-onset, is illustrated. Juvenile-onset HD, in which mutant HTT (mHTT) contains over 60 CAG repeats, is characterized by a decrease in the differentiation of NSCs/NPCs in different *in vitro* models, with the exception of data that were published by Zhang et al. (2019) who discovered premature neurogenesis and neuronal differentiation (the reference is indicated in the figure by an up arrow). Furthermore, NSCs/NPCs exhibited decreases in neurogenesis and disruption in cell organization in juvenile-onset HD. In adult-onset HD, in which mHTT carries equally or more than 39 CAG repeats but not more than 60 repeats, Barnat et al. (2020) reported that mHTT dysregulates neuronal precursors differentiation and directs neurogenesis toward the neuronal lineage. In contrast Conforti et al. (2018) observed an attenuation of NSC differentiation (the reference is indicated in the figure by a down arrow). Additionally, a decrease in cell proliferation, and abnormal cell organization in adult-onset HD NSCs/NPCs *in vitro* and iPSC-derived cortical organoids were reported. References that correspond to the discoveries in juvenile- and adult-onset HD are shown. Under physiological conditions in wildtypes, in GABAergic medium spiny neurons (MSNs), synaptic spine stability is maintained by the SOCE process. In HD MSNs, supranormal synaptic neuronal SOCE (nSOCE) can lead to spine loss and subsequently neurodegeneration. The references that are shown in the figure relate to published data that demonstrate abnormal SOCE in induced pluripotent stem cell (iPSC)-derived MSNs from juvenile- and adult-onset HD and MSNs from the YAC128 transgenic mouse model of HD. Wu et al. (2016) detected an increase in nSOCE in synaptic spines of YAC128 MSNs. The thick red arrow represents the elevation of Ca^2+^ influx via nSOCE. The up or down arrows indicate an increase, a decrease, or dysregulation in proliferation, differentiation, neurogenesis, or cell organization in juvenile- and adult-onset HD.

Till now, the elevation of SOCE was observed in different HD models including MSNs from transgenic YAC128 mice and iPSCs-based GABAergic MSNs from juvenile- and adult-onset HD patient fibroblasts. The involvement of STIM2 in the regulation of the impaired SOCE was confirmed in both a mouse model of HD and juvenile-onset HD iPSCs-derived MSNs. Therefore, one postulation is that the dysregulation of nSOCE underlying HD pathology and neuronal store-operated calcium channels (nSOCs) such as STIM2 could be a novel therapeutic target for HD. Interestingly, STIM2 suppression resulted in attenuation of SOCE in wildtype iPSCs-derived MSNs indicating that STIM2 is responsible for mediating SOC channels in these neurons (Vigont et al., 2021). As shown in Figure 2, elevations of SOCE might underlie the pathology of juvenile- and adult-onset HD. In mature HD MSNs from YAC128 mice, the dysregulation of synaptic spines resulted from abnormal nSOCE mediated by STIM2. The dysregulation of Ca^2+^ signaling pathways was demonstrated in iPSC-derived NPCs and MSNs from HD patients, suggesting its possible role in neurodevelopmental changes in HD.

Further studies using models based on cells from HD patients may bring new findings in understanding HD pathology, therefore it is important to develop techniques to obtain MSNs and cerebral organoids modeling HD. On the other hand, the transplantation of MSN progenitors or iPSC-derived MSNs without a mutation of HTT and with proper Ca^2+^ signaling could result in an advantage in HD patients.

## Modeling HD Using Different Protocols to Obtain Msns

Reprogramming of somatic cells into iPSCs and then differentiating them into MSNs allows the generation of cells that can be a valuable source for research of mechanisms leading to HD pathology. iPSC-based neurons are also crucial because it is impossible to obtain MSNs from living patients. Furthermore, post-mortem samples can be disrupted and unreliable in terms of disease progression and severity. Therefore, an unlimited source of iPSC-based MSNs can be invaluable. Recently, GABAergic MSNs that are based on HD iPSCs were suggested to be used as a platform for personal HD screening (Bezprozvanny and Kiselev, 2017; Vigont et al., 2018).

Human iPSCs can be obtained from somatic cells [(e.g., human fibroblasts, blood cells, keratinocytes from hair, and renal tubular epithelial cells from urine (Raab et al., 2014)], which are transduced by a lentiviral vector with a combination of four reprogramming transcription factors, including octamer-binding transcription factor 3/4 (Oct3/4), SOX2, Kruppel-like factor 4 (Klf4), and c-Myc (Takahashi et al., 2007). Park et al. (2008) were the first who reprogramming fibroblasts from HD patients into iPSCs. The generated iPSC line contained 72 CAG polyglutamine repeats in one allele and 19 in the other (Park et al., 2008). Recently reprogramming of iPSCs with the application of non-integrative methods has become more popular (Schlaeger et al., 2015). In February 2020, The European Bank for induced Pluripotent Stem Cells (EBiSC) and CHDI Foundation, in cooperation with Censo Biotechnologies, generated widely available iPSC lines from HD patients^1^.

However, other cell types are also used to obtain MSNs, including embryonic stem cells that are pluripotent stem cells isolated from the inner cell mass (embryoblasts) of donated blastocysts. When they differentiate into neuronal progenitors, in addition to neurons, they can further transform into astrocytes and oligodendrocytes (Im et al., 2009). The advantage of iPSCs over ESCs during HD stem cell therapy is also evident. In the case of ESCs, allogenic immunological rejection and tumor formation were reported (Im et al., 2009; Liu et al., 2016). NSCs/NPCs are also used as sources of stem cells to generate human HD MSNs (Lin et al., 2015; Golas, 2018).

Various protocols to obtain MSNs from iPSCs have been developed (Zhang et al., 2010; Delli Carri et al., 2013; Arber et al., 2015; Lin et al., 2015; Liu et al., 2016; Nekrasov et al., 2016; Adil et al., 2018; Golas, 2018; Csobonyeiova et al., 2020; Grigor’eva et al., 2020; Xu et al., 2020; Vigont et al., 2021). These protocols may be based on other already established protocols that are modified.

Adult-onset HD iPSCs-based GABAergic MSNs cultures, which were characterized by abnormal Ca^2+^ influx via SOCs, were cultured using the protocol that consists of four steps (Nekrasov et al., 2016). From the start of differentiation to receiving terminally differentiated neurons, more than 60 days elapse. To obtain HD iPSC-based GABAergic MSNs in the induction step, the authors used Noggin, TGF-β RI kinase inhibitor VI (SB-431542), and dorsomorphin to inhibit the bone morphogenetic protein (BMP)/TGF-β pathway and differentiate hPSCs into neuroepithelial cells (Nekrasov et al., 2016). The combined use of Noggin/SB-431542 and dihydrochloride (LDN-193189), referred to as dual SMAD inhibition, was applied in earlier protocols (Delli Carri et al., 2013; Nicoleau et al., 2013). Maintaining the use of Noggin and adding purmorphamine resulted in cell differentiation into lateral ganglionic eminence (LGE) progenitors (Nekrasov et al., 2016). However, most protocols are based on the use of only one of these factors (Delli Carri et al., 2013; Nicoleau et al., 2013; Adil et al., 2018; Grigor’eva et al., 2020). Striatal projection neurons derive especially from the LGE (Reddington et al., 2014). In the next step of the protocol of Nekrasov et al. (2016) neural rosettes were mechanically reconstructed to separate NPCs from other cell types. The self-arranged formation of rosettes by neuroectodermal or neuroepithelial progenitor cells is a morphological signal that neuronal induction has started (Golas, 2018; Comella-Bolla et al., 2020). This is a transition stage to differentiation into a neuronal or glial lineage entry in response to relevant developmental signals (Elkabetz et al., 2008). To differentiate NPCs into mature HD iPSC-based GABAergic MSNs, brain-derived neurotrophic factor (BDNF) and forskolin were used (Nekrasov et al., 2016; Comella-Bolla et al., 2020). Forskolin is a cAMP signal conduction activator and not widely used for MSN terminal differentiation. However, it is a morphogen that is used for the direct differentiation of fibroblasts into cholinergic and glutaminergic neurons and motoneurons (Xu et al., 2020). Dibutyryl-cAMP (db-cAMP) is a cAMP analog that is used more frequently (Zhang et al., 2010; Nicoleau et al., 2013; Victor et al., 2014; Lin et al., 2015; Adil et al., 2018; Grigor’eva et al., 2020). Finally, Nekrasov et al. (2016) obtained HD iPSCs-based GABAergic MSNs cultures that expressed synaptic GABA transporter 1 (GAT1), had dendritic spines, formed synapses, and manifested an HD phenotype, which was more prominent with the culture age.

Grigor’eva et al. (2020) developed a new three-step protocol for human iPSCs differentiation into striatal MSNs. The protocol assumes dual SMAD inhibition to obtain neuroectodermal cells. For this purpose, the authors used SB-431542 and LDN-193189. These small molecules inhibit BMP/TGF-β signaling and induce ventral telencephalic specification, enhanced by the addition of purmorphamine. This combination significantly enhanced neural induction (Grigor’eva et al., 2020). Ventral telencephalic identity induction was also performed using sonic hedgehog (SHH) (Zhang et al., 2010; Delli Carri et al., 2013; Nicoleau et al., 2013; Lin et al., 2015), for which purmorphamine is an agonist (Ma et al., 2012). Ma et al. (2012) showed that 0.65 μM purmorphamine can be the equivalent of 200 ng/ml SHH for generating LGE-like progenitors. As a factor that improves the survival of neurons, bFGF, also known as FGF2 was used (Zhang et al., 2010; Nekrasov et al., 2016; Grigor’eva et al., 2020). In the next stage, subsequent precursors of GABAergic projection MSNs (pMSNs) were cultivated, which was an important attribute of their protocol (Grigor’eva et al., 2020). Finally, the terminal differentiation of cells into MSNs was performed by adding activin A, which promotes the differentiation of cells toward the LGE. Arber et al. (2015) were the first, who proposed the use of activin A as an inducer of LGE phenotype in neuronal precursors from hESCs and hiPSCs in an SHH-independent manner during the derivation of MSNs. This methodology was later used by Fjodorova et al. (2019) who differentiated hPSCs, hESCs, and hiPSCs into HD MSNs. Additionally, both BDNF and ascorbic acid (AA) were supplemented in the media. The high-frequency use of BDNF at the stage of regionalization and maturation indicates that this is an important and necessary factor that allows the generation of MSNs. The obtained cells expressed striatal marker dopamine- and cAMP-regulated neuronal phosphoprotein (DARPP-32), forkhead box protein P2 (FOXP2), and calbindin 1 (CALB1). The authors skipped the stage of the manual collection of rosette-like structures compared with the protocol of Nekrasov et al. (2016), but they observed the formation of these structures (Grigor’eva et al., 2020). They also did not extend co-cultivation on feeder cells compared with Zhang et al. (2010), who cultured iPSCs on irradiation-inactivated mouse embryonic fibroblasts (MEFs). However, Grigor’eva et al. (2020) used Matrigel or poly-D-lysine/laminin-coated plates. They also demonstrated the possibility of pMSNs cryopreservation from day 20 to 180 of differentiation. After reseeding, cell survival was over 95%, and pMSNs were able to differentiate into terminal neurons. Terminally differentiated MSNs were characterized after 58-day pMSNs were obtained, which took 12 days to mature (Grigor’eva et al., 2020).

To obtain juvenile-onset HD iPSCs-derived MSNs, which characterize by abnormal SOCE (Vigont et al., 2021), applied protocol based on double inhibition of the SMAD cascade using SB-431542 and LDN-193189. Next, purmorphamine treatment directed cells into LGE, and further neuronal maturation has been achieved with the application of neurotrophic factors BDNF and GDNF as well as forskolin (Nekrasov et al., 2016; Comella-Bolla et al., 2020). Neuronal maturation took approximately 20 days and differentiated neurons expressed neuronal and striatal markers. However, no visible differences in neuronal morphology and viability between control and mutant cells were detected. Additionally, the protocol described by Vigont et al. (2021) allows cryopreservation of NPCs.

One of the most rapid protocols that involve the transdifferentiation of fibroblasts into MSNs has been recently established (Victor et al., 2014). These authors transduced fibroblasts with lentiviruses to obtain the co-expression of microRNA (miR)-9/9^∗^ and miR-124 (miR-9/9^∗^-124), which play a key role in differentiating NPCs into mature neurons. The induced neurons (iNs) were then transduced with lentivirus to express transcription factors that are involved in development of the striatum, such as chicken ovalbumin upstream promoter (COUP)-transcription factor (TF)-interacting protein 2 (CTIP2), distal-less homeobox 1 (DLX1), DLX2, and myelin transcription factor 1 like (MYT1L). Neuronal culture medium was supplemented with valproic acid (VPA), db-cAMP, BDNF, and retinoic acid (RA). Thirty-five days after transduction, the obtained cells were positive for markers of MSNs, including microtubule-associated protein 2 (MAP2), GABA, and DARPP-32 (Victor et al., 2014). Other reports present that MSNs can also be produced based on the transdifferentiation of somatic cells (Richner et al., 2015; Gascón et al., 2017; Victor et al., 2018).

Wu M. et al. (2018) developed a protocol, called XLSBA, that involves the differentiation of ESCs by replacing protein components with small molecules and adding the γ-secretase inhibitor DAPT to enhance neural differentiation. This protocol allowed the generation of MSNs that can be used as a cell-based therapy for HD patients. To obtain MSNs that expressed appropriate markers and exhibited electrophysiological properties, 21–24 days is needed. The shorter time to obtain MSNs increases the ability to control the conditions and reduce variability between successive neuron generations. Additionally, the restriction of protein elements eliminates difficulties in quality control and reduces costs. In this protocol, the authors replaced Noggin with LDN-1931189 and SB-431542, thereby achieving the effect of dual SMAD inhibition. To inhibit WNT signaling, they used the tankyrase inhibitor (XAV-939). By combining four small molecules (XAV939, LDN-193189, SB-431542, and SAG, the SHH signaling agonist), they obtained precursors of LGE that express the striatal markers CTIP2 and DARPP-32. The only protein factors that were used were BDNF and glial cell line-derived neurotrophic factor (GDNF) for terminal differentiation (Wu M. et al., 2018). Similar to Grigor’eva et al. (2020), they used AA for maturation. Finally, Wu et al. achieved 90% of DARPP-32-positive GABAergic MSNs that are suitable for cell transplantation (Wu M. et al., 2018). Additionally, hESCs were used by several groups as *in vitro* models of HD to understand the mechanisms of neurodegeneration in HD patients (Niclis et al., 2009; Bradley et al., 2011; Dumevska et al., 2016; Xie et al., 2016).

Currently, no protocols are available that can generate MSNs from iNSCs that are obtained directly *in vitro*. The only successful attempts differentiate iPSC-derived NPCs (Lin et al., 2015), HD-iPSC-derived NSCs (Zhang et al., 2010), and striatal human neural stem cell (hNSC) lines (El-Akabawy et al., 2011) into MSNs. However, Al-Gharaibeh et al. (2017) obtained iNSCs from iPSCs (iPSCs-NSCs), which expressed the NSC markers Nestin and SOX2. After intra-striatal transplantation into YAC128 mice, iPSCs-NSCs differentiated into region-specific MSNs. These cells co-expressed a neuronal nuclei (NeuN), which is a marker of mature neurons, and the region-specific marker DARPP-32. Additionally, iNSC-treated mice exhibited the amelioration of motor deficits and an increase in BDNF levels in the striatum (Al-Gharaibeh et al., 2017). Evidence indicates that induced neural stem cells (iNSCs), NSCs, and NPCs do not form teratomas after transplantation in animal models (Gao et al., 2016; Deng et al., 2018). Furthermore, NSCs/NPCs can be induced from somatic cells (Han et al., 2012; Ring et al., 2012; Capetian et al., 2016; Choi and Hong, 2017; Kim et al., 2017). Table 2 summarizes small molecules, inhibitors, transcription factors, and growth factors that are widely used for the differentiation of iPSCs (Zhang et al., 2010; Delli Carri et al., 2013; Arber et al., 2015; Nekrasov et al., 2016; Adil et al., 2018; Comella-Bolla et al., 2020; Grigor’eva et al., 2020; Vigont et al., 2021), ESCs (Nicoleau et al., 2013; Arber et al., 2015; Wu M. et al., 2018), and NPCs (Lin et al., 2015), NSCs (El-Akabawy et al., 2011) and fibroblast transdifferentiation into MSNs (Victor et al., 2014). The table also shows the steps of MSNs development where they are used *in vitro*.

**TABLE 2:** Small molecules, inhibitor, transcription factors, and growth factors used for iPSC, ECS, and NPC differentiation and transdifferentiation.

Substance/Factor	Function	Differentiation step	References
TGF-β RI kinase inhibitor VI (SB-431542)	- BMP/TGF-β signaling inhibition - Enhanced neuronal differentiation - Induction of ventral telencephalic specification - PSC differentiation into neuroepithelial cells	Neural induction	Delli Carri et al., 2013; Nicoleau et al., 2013; Arber et al., 2015; Adil et al., 2018; Comella-Bolla et al., 2020; Grigor’eva et al., 2020; Vigont et al., 2021
		Patterning	Delli Carri et al., 2013
Noggin	- BMP/TGF-β signaling inhibition - PSC differentiation into neuroepithelial cells	Neural induction and patterning	Delli Carri et al., 2013; Nicoleau et al., 2013; Nekrasov et al., 2016
Purmorphamine	- BMP signaling inhibition - Enhanced neuronal differentiation	Neural induction	El-Akabawy et al., 2011; Adil et al., 2018
		Patterning	El-Akabawy et al., 2011; Nekrasov et al., 2016; Adil et al., 2018; Grigor’eva et al., 2020; Vigont et al., 2021
		Maturation	El-Akabawy et al., 2011
Brain-derived neurotrophic factor	- Neuronal survival factor - Increase the proportion of DARP-32-positive neurons	Patterning	Zhang et al., 2010; Delli Carri et al., 2013; Lin et al., 2015; Adil et al., 2018; Grigor’eva et al., 2020
		Maturation	El-Akabawy et al., 2011; Delli Carri et al., 2013; Nicoleau et al., 2013; Victor et al., 2014; Arber et al., 2015; Lin et al., 2015; Nekrasov et al., 2016; Wu M. et al., 2018; Comella-Bolla et al., 2020; Grigor’eva et al., 2020; Vigont et al., 2021
Dihydrochloride (LDN-193189)	- BMP/TGF-β signaling inhibition - Induction of ventral telencephalic specification	Neural induction	Arber et al., 2015; Wu M. et al., 2018; Comella-Bolla et al., 2020; Grigor’eva et al., 2020; Vigont et al., 2021
Sonic hedgehog	- Induction of forebrain neurogenesis - Promote differentiation into DARPP-32-positive neurons	Patterning	Zhang et al., 2010; El-Akabawy et al., 2011; Delli Carri et al., 2013; Nicoleau et al., 2013; Lin et al., 2015
Basic fibroblast growth factor	- Neuronal survival factor	Neural induction	Al-Gharaibeh et al., 2017
		Patterning	Zhang et al., 2010; Nekrasov et al., 2016; Grigor’eva et al., 2020; Vigont et al., 2021
Glial cell line-derived neurotrophic factor	- Neuronal survival factor	Maturation	Arber et al., 2015; Lin et al., 2015; Adil et al., 2018; Wu M. et al., 2018; Vigont et al., 2021
Dorsomorphin	- BMP signaling inhibition - Enhanced neuronal differentiation - Induction of ventral telencephalic specification - PSC differentiation into neuroepithelial cells	Neural induction	Arber et al., 2015; Nekrasov et al., 2016; Grigor’eva et al., 2020
Forskolin	- cAMP pathway activator	Patterning and maturation	Nekrasov et al., 2016
		Maturation	Comella-Bolla et al., 2020; Vigont et al., 2021
Activin A	- TGF-β/BMP signaling pathway activation - Induction of forebrain neurogenesis - Differentiation in the lateral ganglionic eminence - Promote differentiation into DARPP-32-positive neurons	Patterning and maturation	Arber et al., 2015; Grigor’eva et al., 2020
Dibutyryl-cAMP	- Cell-permeable analog of cAMP - Enhance the output of depolarized neurons - Stimulate GABAergic MSNs neurogenesis	Maturation	Zhang et al., 2010; El-Akabawy et al., 2011; Nicoleau et al., 2013; Victor et al., 2014; Lin et al., 2015; Adil et al., 2018; Grigor’eva et al., 2020
Ascorbic acid	- Formation and maintenance of the neural microenvironment - Neuroprotective	Maturation	Wu M. et al., 2018; Comella-Bolla et al., 2020; Grigor’eva et al., 2020
ROCK inhibitor (Y-27632)	- Stem cell survival factor	Neural induction	Compagnucci et al., 2016; Comella-Bolla et al., 2020
		Patterning	Zhang et al., 2010; Delli Carri et al., 2013; Lin et al., 2015; Compagnucci et al., 2016; Grigor’eva et al., 2020
Valproic acid; Valpromide	- Stimulation of GABAergic MSN neurogenesis	Maturation	Zhang et al., 2010; El-Akabawy et al., 2011; Nicoleau et al., 2013; Victor et al., 2014
Neurotrophin-3	- Neurotrophic factor	Maturation	Victor et al., 2014
Retinoid acid	- Induce GABAergic differentiation	Patterning and maturation	El-Akabawy et al., 2011; Victor et al., 2014
WNT inhibitor dickkopf 1	- WNT signaling inhibition	Patterning	Zhang et al., 2010; El-Akabawy et al., 2011; Delli Carri et al., 2013; Nicoleau et al., 2013; Adil et al., 2018
Insulin	- Proliferation improvement	Patterning	Delli Carri et al., 2013; Shahbazi et al., 2019
		Maturation	Delli Carri et al., 2013; Lin et al., 2015; Shahbazi et al., 2019
Insulin-like growth factor 1	- Synapse formation	Maturation	Lin et al., 2015; Nieto-Estévez et al., 2016; Adil et al., 2018
Tankyrase inhibitor (IWR1)	- WNT signaling inhibition	Neural induction and patterning	Comella-Bolla et al., 2020
Tankyrase inhibitor (XAV-939)	- WNT signaling inhibition	Patterning	Wu M. et al., 2018
			

**FIGURE 3:**
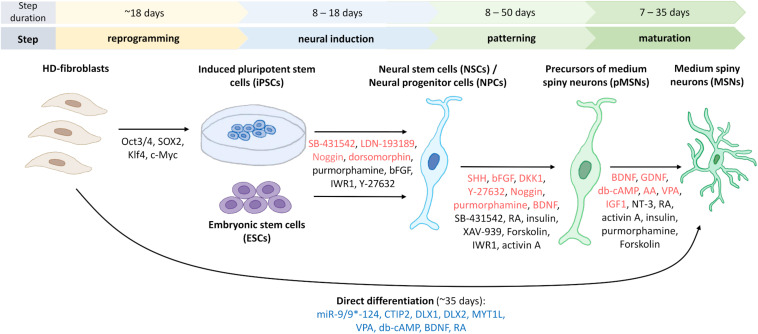
Generation of human striatal MSNs from fibroblasts and stem cells. To reprogram fibroblasts into induced pluripotent stem cells (iPSCs), four transcription factors are used to transduce cells from HD patients (Victor et al., 2018). Indicated in red are the most commonly used factors for neural induction of iPSCs or embryonic stem cells (ESCs) to obtain neural stem cells (NSCs)/neural progenitor cells (NPCs), and for differentiation of progenitors and mature GABA-ergic medium spiny neurons, pMSNs and MSNs, respectively. Marked in black are factors used for neural induction or differentiation only in one or two protocols, which are discussed in the manuscripts. For direct differentiation, microRNA-9/9*-124, and other factors that are indicated in blue were used (Victor et al., 2014). The duration of each of the differentiation steps differs according to the various protocols. Figure summarizes fourteen protocols describing the generation of human striatal MSNs from iPSCs (Zhang et al., 2010; Delli Carri et al., 2013; Arber et al., 2015; Nekrasov et al., 2016; Adil et al., 2018; Comella-Bolla et al., 2020; Grigor’eva et al., 2020; Vigont et al., 2021), fibroblasts (Victor et al., 2014), ESCs (Nicoleau et al., 2013; Arber et al., 2015; Wu M. et al., 2018), NSCs (El-Akabawy et al., 2011), and NPCs (Lin et al., 2015). Using bFGF and neural media for culturing, it is possible to differentiate iPSCs to induced neural stem cells (iNSCs) and then transplant them into the striatal region of the mouse brain where they differentiate into MSNs (not shown) (Al-Gharaibeh et al., 2017).

Finally, Figure 3 shows a schematic representation of the protocols that are established to obtain MSNs *in vitro* from different cell sources.

## Modeling HD Using Brain Organoids

**FIGURE 4:**
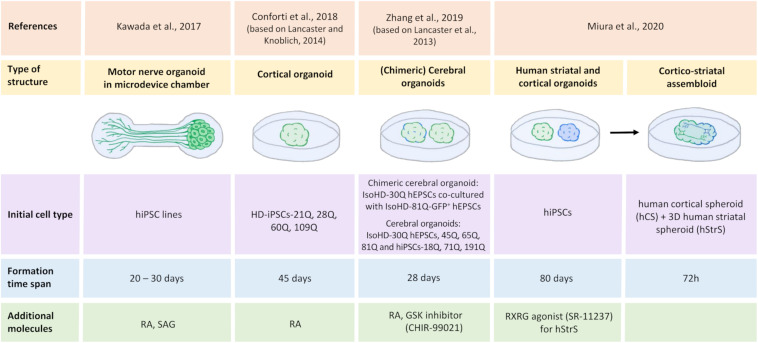
Generation of human iPSC-derived three-dimensional brain structures modeling HD. All illustrated HD organoids, including two region-specific brain organoids that consist of the three-dimensional (3D) human striatal spheroid (hStrS) and human cortical spheroid (hCS) that were used to create the assembloid by Miura et al. (2020), originated from induced pluripotent stem cells (iPSCs). Cortical organoids (Conforti et al., 2018; Zhang et al., 2019) and the chimeric organoids (Zhang et al., 2019) represent 3D cell models of Huntington’s disease. Motor nerve organoids extended axons as a result of their culture in the special chamber with microchannels (Kawada et al., 2017). The organoid formation time differs depending on the protocol. Neural differentiation inducers were used in the organoids formation process: retinoic acid (RA) (Kawada et al., 2017; Conforti et al., 2018; Zhang et al., 2019), retinoid X receptor γ (RXRG) agonist, SR-11237 (Miura et al., 2020), glycogen synthase kinase (GSK) inhibitor, CHIR-99021 (Zhang et al., 2019), and sonic hedgehog signaling agonist (SAG) (Kawada et al., 2017).

By developing 3D culture methods based on the ability of iPSCs to self-organize and gene editing, the first organoids could be created, which can mimic brain tissue architecture. The complexity and characterization of their structures allow them to be used as an efficient and suitable model for drug and toxicity testing as a substitute for animal models (Csobonyeiova et al., 2020). It is now possible to create different types of organoids, including midbrain, cerebral, and hippocampal organoids, that can be used to understand disease development and progression (Lancaster and Knoblich, 2014; Yadav et al., 2020). Advances in obtaining 3D organoids that may serve as a model for studying HD has been made in recent years. Figure 4 shows schematic methods of culturing iPSC-derived 3D organoids and assembloids for modeling HD.

Huntington’s disease is characterized by motor dysfunction and incoordination. Kawada et al. (2017) created motor nerve organoids that derived from human iPSCs. Authors first differentiated human-induced pluripotent stem cells (hiPSCs) into spinal motor neurons. They then placed these neurons in the vessel with low adhesion. Under these conditions, the cells began to form spheroids. The spheroids were then moved into a culture microdevice, which was a special chamber for the spheroid that turns into a microchannel that ends in the chamber to axon terminals. Finally, after 20–30 days of culture in the microdevice, the spheroid-forming neurons spontaneously extended the axons. In this way, nerve organoids were formed that consisted of a unidirectional fascicle and neural spheroid (Kawada et al., 2017). The resulting motor nerve organoids may be a good model of HD to study disturbances in Ca^2+^ homeostasis and provide insights into the projection of neural networks and synaptic plasticity.

Another attempt to model HD was made by Conforti et al. (2018) who investigated HD-iPSC-derived striatal and cortical neurons and HD-iPSC-based 3D cerebral organoids that corresponded to adult- and juvenile-onset HD. Selected HD-iPSC lines that were used to generate neurons or organoids carried 60, 109, and 180 CAG repeats, whereas the control lines carried 21, 28, and 33 CAG repeats in HTT. To obtain MSNs, both SB-431542 and LDN-193189 were used for neural induction, similar to MSNs that were obtained previously (Shi et al., 2012; Comella-Bolla et al., 2020; Grigor’eva et al., 2020), whereas striatal differentiation was performed according to the Delli Carri et al. (2013) protocol. Cortical projection neurons were differentiated using the three-stage protocol (Shi et al., 2012). To obtain 3D cortical organoids, a methodology describing cerebral organoids generation was applied (Lancaster and Knoblich, 2014). According to the hanging drop method, on day 3, Conforti et al. (2018) obtained HD-iPSC-derived spheroids. After being placed on the horizontal shaker, spheroids formed embryonic bodies (EBs). The EBs were then neuroectodermally differentiated using neural induction medium. The resulting cell aggregates were placed in drops of Matrigel with the medium for neural differentiation. In the next stage, neuroepithelial bud expansion and promotion occurred for further development into the cortical part of the brain (Conforti et al., 2018). Conforti et al. (2018) established that mHTT affects human neurodevelopment through the negative regulation of striatal and cortical specification in both juvenile- and adult-onset HD iPSC-derived cerebral organoids where a decrease in neuronal differentiation and cell disorganization were detected. They found that mHTT that carried longer CAG expansions led to the total failure of neuroectodermal acquisition, whereas cells that contained shorter CAG repeats were characterized by several abnormalities in neural rosette formation and improper cortical organoid cytoarchitecture. Moreover, HD lines exhibited a slower exit from pluripotency in a CAG-dependent manner and defects in cortical and striatal progenitors, neuronal specification, and terminal neuronal maturation. Analyses of gene expression in HD organoids confirmed that they overlapped with the immature ventricular zone/SVZ, whereas control organoids corresponded to mature human fetal cortical areas. Huntington’s disease organoids also exhibited a reduction of the expression of genes that are related to the regulation of neuronal migration and differentiation. Additionally, the downregulation of mHTT and inhibition of its effector metalloprotease ADAM10 rescued defects in neuronal induction and striatal differentiation in HD lines. These data confirmed the role of mHTT in abnormal neurodevelopment in HD (Conforti et al., 2018).

Additionally, Zhang et al. (2019) investigated the correlation between prolonged CAG repetitions (polyQ tail) of huntingtin and control, juvenile- and adult-onset HD and the influence on early neurodevelopment in HD hESC-derived cerebral organoids. The authors used isogenic HD ESCs with 30, 45, 65, and 81 CAG repeats (IsoHD hESCs 30Q, 45Q, 65Q, and 81Q, respectively) and HD hiPSCs with 18Q, 71Q, and 191Q to create organoids. They also generated chimeric cerebral organoids from co-cultures of IsoHD hESCs 81Q labeled with a green fluorescent protein and IsoHD hESCs 30Q. The protocol that was used by Zhang et al. (2019) was based on Lancaster et al. (2013) protocol for the induction of cerebral organoid formation. hESCs were first used to create EBs, after which deposition in Matrigel formed a neuroectoderm with a neuroepithelium (Zhang et al., 2019). To obtain shaping toward the forebrain, a glycogen synthase kinase (GSK) inhibitor, CHIR-99021 was added to the medium. On day 28 of culture, the obtained organoids resembled the human brain, corresponding to 2–3 months of human brain development *in vivo*. Expression of the cortical and forebrain markers CTIP2 and forkhead box G1 (FOXG1), respectively, was detected in these organoids. Additionally, the presence of radial (neuroepithelial) structures that expressed paired box protein 6 (PAX6) was detected (Zhang et al., 2019). In contrast to Conforti et al. (2018), Zhang et al. (2019) found premature neurogenesis and neuronal differentiation in IsoHD-81Q cerebral organoids corresponding to juvenile-onset HD. Zhang et al. (2019) showed that ventricular zone-like neuroepithelial progenitor layer expansion was blunted by an increase in the number of CAG repeats in mHTT because of premature neurogenesis in these organoids. Furthermore, impairments in cell cycle regulatory processes and an increase in activity of an upstream regulator of the cell cycle (i.e., the ataxia telangiectasia mutated [ATM]-p53 pathway) were identified, which might be responsible for premature neuronal differentiation. Upon the application of ATM antagonists, the partial rescue of blunted neuroepithelial progenitor expansion was detected in HD organoids (Zhang et al., 2019). The authors proposed that the length of HTT polyQ tails controls the ratio between NPC proliferation and differentiation in the early stages of nervous system development (Zhang et al., 2019).

Finally, Miura et al. (2020) were the first to develop a protocol to generate human 3D brain organoids that resemble the LGE, corresponding to the striatum during development. To obtain these organoids, hiPSCs were used. Neuronal differentiation was induced using SMAD and WNT modulators and the application of activin A, which promotes differentiated cells in the striatal direction. To obtain LGE patterning, transcriptomic research was performed. Miura et al. found that the gene that encodes retinoid X receptor γ (RXRG) was highly expressed in the early developing striatum. By adding the RXRG agonist SR-11237, they increased the proportion of CTIP2-positive cells, which are a marker of early striatal cells. Moreover, they found that neurons that were obtained from human striatal spheroids (hStrSs) had electrophysiological characteristics of striatal MSNs (Miura et al., 2020). The authors assembled hStrSs with human cortical spheroids (hCSs) shaped like the cerebral cortex to form cortico-striatal assembloids. They found that the resulting structure sent axonal projection neurons into hStrSs and functionally connected with MSNs. Lastly, cortico-striatal assembloids were used to examine defects in cortico-striatal circuits in patients with 22q13.3 deletion syndrome (22q13.3DS) (Miura et al., 2020). They may also serve as a model to investigate cortico-striatal circuits or transneuronal cortico-striatal spreading of mHTT in HD (Pecho-Vrieseling et al., 2014; Miura et al., 2020).

The further development of 3D organoids that model HD is needed. These models will likely provide novel insights into the regulation of synaptic plasticity and maturation of striatal and motor neurons (Chang et al., 2020). Different signal pathways, including Ca^2+^ signaling, can also be studied using these models. Although 3D organoid technology still has many obstacles to overcome, it will certainly contribute to our understanding of complex processes of neurodevelopment, disease progression, and pathogenesis. Patient-derived organoids may also be useful for generating personalized models of disease and thus personalized HD treatment strategies.

## Application of Stem Cell in HD Therapy

Although a few compounds have been shown to stabilize elevations of SOCE in HD models, future studies are necessary to evaluate their potential for HD therapy (Czeredys, 2020). No treatments for HD are currently available, but this disease may be a good candidate for cell replacement therapy because it is characterized by the relatively focal loss of MSNs that is caused by a mutation of HTT (Rosser and Bachoud-Lévi, 2012). It was suggested that mHTT disrupts Ca^2+^ signaling in MSNs and those changes could be a cause of HD progression (Raymond, 2017; Pchitskaya et al., 2018; Czeredys, 2020). Therefore transplantation of MSN progenitors without a mutation of HTT appears to be beneficial for HD patients. To achieve regeneration in HD, donor cells should have the ability to be precisely differentiated into MSNs, and these cells should be functionally active (Precious and Rosser, 2012). The application of donor cells that are derived from the whole ganglionic eminence (WGE) in the ventral telencephalon in the fetal brain is a very promising approach (Döbrössy and Dunnett, 2003; Mazzocchi-Jones et al., 2009; Pauly et al., 2012). This brain region is the origin of cells that are committed to striatal MSN phenotypes (Deacon et al., 1994; Olsson et al., 1998; Straccia et al., 2016). Several groups reported that the transplantation of developing MSNs into the degenerating striatum in different animal models of HD led to functional recovery and ameliorated motor and cognitive deficits (Björklund et al., 1994; Kendall et al., 1998; Palfi et al., 1998; Brasted et al., 1999; McLeod et al., 2013; Schackel et al., 2013; Paganini et al., 2014). Similar recovery in rats grafted with human whole ganglionic eminence (hWGE) compared to rat WGE was shown, with the additional benefit of the hWGE stabilizing performance on the adjusting steps test (Lelos et al., 2016).

Evidence from initial clinical trials showed that human fetal-derived grafts that were transplanted into the striatum of three out of five HD patients survived and significantly improved motor and cognitive function over an approximately 6-year period (Bachoud-Lévi et al., 2000, 2006). Additionally, enhanced fluorodeoxyglucose positron emission tomography showed that implanted cells were able to integrate into striatal neural circuits and created functional connections with cortical regions (Gaura et al., 2004). Other pilot studies performed by different groups reported some beneficial effects for HD patients who received fetal neurografts into the striatum (Hauser et al., 2002; Rosser et al., 2002; Gallina et al., 2008; Reuter et al., 2008; Barker et al., 2013). In longer-term follow-up data from these initial trials, where a total of 51 patients have been transplanted using human fetal cells, signs of long-term efficacy have been reported in 4 out of 30 patients for which clinical data and follow-up are available (Bachoud-Lévi, 2017). Interestingly, it was shown in post mortem analysis of HD patients that grafts might survive at least 10 years after transplantation (Bachoud-Lévi, 2009; Cicchetti et al., 2009). Preliminary stem cell transplants provided the basis for the largest fetal cell transplant trial Multicentric Intracerebral Grafting in Huntington’s Disease (MIG-HD), which involved 45 HD patients. It was initiated to investigate the efficacy of transplantation as well as its applicability in a multicenter approach. However, the lack of clinical benefit was found in these trial, what might be related to graft rejection. Total motor score at month 32 did not differ between grafted and control groups. In 40% of the transplanted patients, antihuman leucocytes antigen antibodies were found (Krystkowiak et al., 2007; Bachoud-Lévi and on behalf the Multicentric Intracerebral Grafting in Huntington’s Disease Group., 2020). In the German branch of MID-HD, where additional 22 patients were transplanted no clinical benefit was detected (Capetian et al., 2009; Krebs et al., 2011; Lopez et al., 2014). MID-HD studies failed to identify the methodology to reliably reproduce pilot results, although procedural improvements have significantly increased the safety of surgery (Bachoud-Lévi et al., 2020).

Limitations of human fetal tissue and other aspects that make their use difficult (Precious et al., 2017; Bachoud-Lévi et al., 2020) have prompted the need to search for alternative donor cell sources that can be cultured, expanded, and reprogrammed into MSNs before their therapeutic use (Csobonyeiova et al., 2020). ECs and adult iPSCs appear to be a good cell source for replacement therapy since they can be directed to MSN-like cell fates. However, it is difficult to obtain a genuine MSN fate, and further advances are needed to achieve suitable cells for replacement therapy for HD (Li and Rosser, 2017; Golas, 2018; Bachoud-Lévi et al., 2020). Interestingly, hWGE iPSC-derived MSNs that shared fundamental characteristics with hWGE-derived MSNs expect their methylation profiles, differentiated both *in vitro* and following transplantation into an HD model, therefore they might be useful as an alternative cell source for cell replacement therapy (Choompoo et al., 2020). Using iPSCs directly in cell therapy is still challenged because human clinical trials that used iPSCs found unexpected mutations that were caused by the reprogramming technique. Therefore, further research is needed to overcome this critical issue and make cell therapy more feasible (Yoshihara et al., 2017). Recently, hiPSC-derived NPCs that were transplanted into the neonatal mouse striatum differentiated into MSNs and successfully integrated into the host’s brain circuitry without teratoma formation (Comella-Bolla et al., 2020). Moreover, *in vitro* differentiated human striatal progenitors, which were transplanted into the striatum from a rat model of HD matured and integrated into host circuits (Besusso et al., 2020). Additionally, other cell types, such as mesenchymal stem cells, are being administrated intravenously to HD patients (Salado-Manzano et al., 2020).

## Concluding Remarks

Ca^2+^ signaling via store-operated Ca^2+^ channels may control both physiological and pathological processes in neuronal cells. Here, we discussed the key role of SOCE in the regulation of NSC proliferation, differentiation, and neurogenesis. Several SOCE components play a crucial role in these processes, including STIM proteins and Orai and TRPC channels. Elevations of Ca^2+^ influx via SOC channels, mediated by an increase in STIM2 expression, was observed in HD transgenic mice and caused the dysregulation of dendritic spines in HD MSNs. Interestingly, the elevation of SOCE was also detected in iPSC-based MSNs that were obtained from both juvenile- and adult-onset HD patient fibroblasts. In juvenile-onset HD iPSC-based MSNs elevated expression of STIM2 underlies the excessive Ca^2+^ entry through SOCs in HD pathology. Both neurodevelopmental alterations and Ca^2+^ signaling dysregulation were detected in iPSC-derived MSNs from juvenile HD patients. Furthermore, mHTT was shown to alter human neurodevelopment in adult-onset HD. mHTT was also recently suggested to compromise neurodevelopmental pathways, which can disturb synaptic homeostasis and boost the susceptibility of neurons to the pathological consequences of expanded polyglutamine repeats during HD progression (HD iPSC Consortium., 2017). The involvement of abnormal nSOCE in MSNs from juvenile- and adult-onset HD patients could be concerned with pathological changes that are observed in these patients. The various defects in neurodevelopment that were observed in juvenile- and adult-onset HD *in vitro* models may depend on the length of CAG repeats in mHTT. Whereas the severity of SOCE alterations did not depend on the length of CAG repetitions in different HD onsets. Only in MSNs derived from juvenile-onset HD fibroblasts upregulation of Ca^2+^ sensor STIM2 was shown to contribute to SOCE dysregulation. Considering impairments in Ca^2+^ homeostasis and the dysregulation of other signaling pathways by mHTT, neuronal progenitor cells or differentiated neurons without a mutation of HTT could be grafted to replace degenerated MSNs in HD patients. These therapies could serve as a promising treatment strategy to delay the progression of HD, but further research is needed (Glaser et al., 2019). Therefore, recent progress in the *in vitro* differentiation of MSNs that are derived from different cell sources is essential for HD patients. Additionally, establishing both iPSC-derived HD MSN cultures and HD brain organoids *in vitro* will help us understand the complexity of HD pathology and possible treatment strategies.

## Author Contributions

MC conceived, designed, and wrote the manuscript. EL wrote the manuscript and drew the figures. Both authors contributed to the article and approved the submitted version.

## Conflict of Interest

The authors declare that the research was conducted in the absence of any commercial or financial relationships that could be construed as a potential conflict of interest.

## Footnotes

1 cells.ebisc.org

## Glossary

22q13.3DS, 22q13.3 deletion syndrome; 3D, three-dimensional; A2B5+ NPCs, NPCs with neural cell surface antigen of A2B5; AA, ascorbic acid; ATM, ataxia telangiectasia mutated; BDNF, brain-derived neurotrophic factor; bFGF, basic fibroblast growth factor; BMP, bone morphogenetic protein; Ca^2+^, calcium; CALB1, calbindin 1; cAMP, cyclic adenosine monophosphate; CHIR-99021, GSK inhibitor; CRAC, Ca^2+^ release-activated Ca^2+^; CREB, cAMP-response element-binding protein; CTIP2, chicken ovalbumin upstream promoter (COUP)-transcription factor (TF)-interacting protein 2; DARPP, dopamine- and cAMP-regulated neuronal phosphoprotein; db-cAMP, dibutyryl-cAMP; DKK1, dickkopf 1; DLX, distal-less homeobox; DPSCs, dental pulp stem cells; EBiSC, European Bank for induced Pluripotent Stem Cells; EBs, embryonic bodies; ECs, embryonic cells; EGF, epidermal growth factor; ER, endoplasmic reticulum; ErbB-4, Erb-B2 receptor tyrosine kinase 4; ESCs, embryonic stem cells; FGF, fibroblast growth factor; FOXG1, forkhead box protein G1; FOXP2, forkhead box protein P2; Fura-2AM, Fura-2-acetoxymethyl ester; GABA, γ-aminobutyric acid; GAT1, GABA transporter 1; GDNF, glial cell line-derived neurotrophic factor; GE, ganglionic eminence; GSK, glycogen synthase kinase; hCSs, human cortical spheroids; HD, Huntington’s disease; hESCs, human embryonic stem cells; hiPSCs, human-induced pluripotent stem cells; hNPCs, human neural progenitor cells; hNSC, human neural stem cell; hStrSs, human striatal spheroids; HTT, huntingtin; hWGE, human whole ganglionic eminence; I_CRAC_, Ca^2+^ release-activated Ca^2+^ current; IGF1, insulin-like growth factor 1; iNs, induced neurons; iNSCs, induced neural stem cells; IP3R1, inositol-1,4,5-triphosphate receptor 1; iPSCs, induced pluripotent stem cells; iPSCs-NSCs, iNSCs from iPSCs; I_SOC_, store-operated Ca^2+^ current; isogenic HD ESCs, IsoHD hESCs; IWR1, tankyrase inhibitor; Klf4, Kruppel-like factor 4; KO, knockout; LDN-193189, dichydrochloride; LGE, lateral ganglionic eminence; MAP2, microtubule-associated protein 2; MEFs, mouse embryonic fibroblasts; mESCs, mouse ESCs; mHTT, mutant HTT; MIG-HD, multicentric intracerebral grafting in Huntington’s Disease; miR, microRNA; MSNs, γ-aminobutyric acid (GABA)-ergic medium spiny neurons; MYT1L, myelin transcription factor 1 like; NeuN, neuronal nuclei; NFAT, nuclear factor of activated T cells; NGF, nerve growth factor; NMDAR, *N*-methyl-D-aspartate receptor; NPCs, neural progenitor cells; NSCs, neural stem cells; nSOCE, neuronal store-operated Ca^2+^ entry; nSOCs, neuronal store-operated calcium channels; NT-3, neurotrophin-3; OCT3/4, octamer-binding transcription factor-3/4; PAX6, paired box protein 6; pMSNs, precursors of (GABA)-ergic MSNs; polyQ, polyglutamine residues; RA, retinoic acid; RNAseq, RNA sequencing; ROCK, Rho-associated protein kinase; RXRG, retinoid X receptor gamma; SAG, SHH signaling agonist; SB-431542, TGF-β RI kinase inhibitor VI; SCs, stem cells; SEPT7, septin 7; SGZ, subgranular zone; SHEDs, stem cells from human exfoliated deciduous teeth; SHH, sonic hedgehog; SKF-96365, SOCE inhibitor; SOCE, store-operated Ca^2+^ entry; SOX2, sex determining region Y-box2; SR-11237, RXRG agonist; STIMs, stromal interaction molecule proteins; SVZ, subventricular zone; TGF-β, transforming growth factor beta; TRPC, transient receptor potential canonical; VGCC, voltage-gated Ca^2+^ channel; VPA, valproic acid; WGE, whole ganglionic eminence; XAV-939, tankyrase inhibitor; Y-27632, ROCK inhibitor; YM-58483, SOCE inhibitor.
